# Concurrent inhibition of FAK/SRC and MEK overcomes MEK inhibitor resistance in Neurofibromatosis Type I related malignant peripheral nerve sheath tumors

**DOI:** 10.3389/fonc.2022.910505

**Published:** 2022-07-29

**Authors:** Yihui Gu, Chengjiang Wei, Manhon Chung, Haibo Li, Zizhen Guo, Manmei Long, Yuehua Li, Wei Wang, Rehanguli Aimaier, Qingfeng Li, Zhichao Wang

**Affiliations:** ^1^ Department of Plastic and Reconstructive Surgery, Shanghai Ninth People’s Hospital, Shanghai Jiao Tong University School of Medicine, Shanghai, China; ^2^ Department of Plastic Surgery, The Third Xiangya Hospital, Central South University, Changsha, China; ^3^ Department of Pathology, Shanghai Ninth People’s Hospital, Shanghai Jiao Tong University School of Medicine, Shanghai, China

**Keywords:** neurofibromatosis type I, MPNST, MEK, drug resistance, integrins, FAK (focal adhesion kinase)

## Abstract

Malignant peripheral nerve sheath tumors (MPNST) are aggressive soft-tissue sarcomas which lack effective drugs. Loss of the RAS GTPase-activating protein NF1 and subsequent overactivation of mitogen-activated protein kinase kinase (MAPK) signaling exist nearly uniformly in MPNST, making MAPK inhibition a promising therapeutic intervention. However, the efficacy of MEK inhibitor (MEKi) monotherapy was limited in MPNST and the relative mechanisms remained largely unexplored. In this study, we generated three MEKi-resistant cell models and investigated the mechanisms of MEKi resistance using high-throughput transcriptomic sequencing. We discovered that cell apoptosis and cell cycle arrest induced by MEKi were rescued in MEKi-resistant cells and the upregulation of LAMA4/ITGB1/FAK/SRC signaling conferred resistance to MEKi. In addition, concurrent inhibition of MAPK signaling and FAK/SRC cascade could sensitize MPNST cells to MEKi. Our findings provide potential solutions to overcome MEKi resistance and effective combination therapeutic strategies for treating MPNSTs.

## Introduction

Malignant peripheral nerve sheath tumors (MPNST) are rare soft tissue sarcomas that affect approximately 10% of patients with neurofibromatosis type 1 (NF1), causing severe organ damage and high morbidity (1–4). The only effective therapeutic approach is extended resection with a sufficiently wide margin (3). Unfortunately, many patients lose the chance for surgical resection at the time of diagnosis due to the location or early metastasis of the tumor, and there is no targeted therapy available in the clinic (5). A lack of effective curation makes MPNST the leading cause of early death in adults with NF1 (6). Therefore, it is of great importance to accelerate the development of targeted therapies to improve the prognosis of MPNST patients.

The genomic characterization of MPNST cohorts revealed genes that are dysregulated in MPNSTs, including NF1, CDKN2A, TP53, EED and SUZ12 (7–10). As the most frequent genetic alteration in MPNSTs, inactivation of NF1 leads to the aberrant amplification of Ras and downstream mitogen-activated protein kinase (MAPK) oncogenic signaling, suggesting that this pathway is a potential therapeutic target for MPNSTs. MAPK inhibition using MEK inhibitor (MEKi) has been proven to be a promising strategy for RAS-driven tumors such as melanoma and non–small cell lung cancer (11–13). MEKi selumetinib has been also approved for use in plexiform neurofibroma (pNF), the precursor lesion of MPNSTs (14, 15). However, the efficacy of MEKi monotherapy was relatively limited in MPNSTs and the underlying mechanisms remain largely unexplored, which hinders the implications of MEK inhibitors (MEKis) in MPNST therapy.

Despite the limited effectiveness of MEKis, exploring key mechanisms of MEKi resistance could suggest targets for combinational strategies that hold promise to improve their therapeutic effects. Various mechanisms of MEKi resistance have been elucidated in previous studies (16–21), including kinome reprogramming, tumor microenvironment alterations, and activation of resistance pathways including PI3K/AKT, MAPK, and STAT3 (22–24). In MPNSTs, the research on the mechanisms underlying MEKi resistance was limited and mainly focused on the reactivation of receptor tyrosine kinases (RTK). Although various RTKs that contributed to MEKi resistance have been identified, including MET, PDGFR, and ALK, the development of combination therapy still faces great challenges due to the absence of a commonly dysregulated RTK (25, 26). These findings highlighted the complexity of drug-resistance mechanisms in MEK-targeted therapy, which motivated us to systematically explore the potential mechanisms of MEKi resistance in MPNSTs.

In this study, we established MEKi-resistant MPNST cell models and investigated the mechanism of MEKi resistance using high-throughput transcriptomic sequencing, aiming to improve the efficacy of MEKis in the treatment of MPNSTs. We discovered that the upregulation of LAMA4/ITGB1/FAK/SRC axis led to the reactivation of MAPK pathway, which played a crucial role in MEK inhibitor resistance. We also confirmed that targeting the FAK/SRC cascade could enhance the response of resistant and sensitive MPNST cells to MEKis, providing a potential therapeutic strategy for MPNST therapy.

## Materials & methods

### Cell lines and reagents

MPNST cell lines S462, S462TY, and ST8814 were kindly granted by Prof. Vincent Keng and Prof. Jilong Yang. MPNST cell lines were maintained in high-glucose DMEM supplemented with 10% fetal bovine serum (FBS) and 1% penicillin/streptomycin in a 37°C, 5% CO_2_ incubator. All the cell lines were tested mycoplasma negative every 3 months. Verification of cell lines was confirmed by Short Tandem Repeat DNA profiling (Applied Biological Materials Inc., Canada).

Reagents and antibodies used in this article are described in **Supplementary Table S1**.

### Generation of drug-resistant cell lines

To generate MEKi-resistant cell lines, the parental NF1-MPNST cell line S462 was induced by conventional continuous exposure to trametinib, TAK-733, and selumetinib in a dose-stepwise increment for five months (with a change in medium three times per week).

### Cell line-based assays

A Cell Counting Kit-8 (CCK-8) assay was implemented to assess cell proliferation and cytotoxicity. a total of 3*10^3^ cells per well were seeded and treated with 0.1% DMSO or the indicated drugs. After 72 h, 10 μL CCK-8 solution (Dojindo, Japan)dispersed in 90 μL DMEM was added per well to measure the450 nm OD value after a 2 h incubation. Percentage cell viabilitywas calculated as 100% × (OD of drug-treated cells - OD ofbackground control)/(OD of untreated cells - OD of backgroundcontrol). The IC50 of indicated drugs was calculated by Prism8.4.0 using [inhibitor] vs. normalized response – Variable slopeanalysis. Potential synergistic or additive effects were quantifiedusing the software CompuSyn (Cambridge, UK) as previouslydescribed (27, 28). Combination index (CI) values calculated,where CI < 0.9, 0.9–1.1, and > 1.1 indicate synergism, additiveeffect, and antagonism, respectively.

Annexin V–FITC and propidium iodide (PI) assays were implemented to detect cell apoptosis. MPNST cells were seeded into 6-well culture plates and treated with DMSO or the indicated inhibitors for 24 h. Afterwards, MPNST cells were stained with annexin V–FITC and PI (BD-Pharmingen, United States) at room temperature in the dark for 15 minutes, followed by analysis using a flow cytometer (Beckman Coulter, Shanghai) equipped with CytExpert software (Beckman Coulter, Shanghai).

Cell cycle assays were implemented to monitor cell cycle. MPNST cells were seeded into 6-well culture plates and treated with DMSO or the indicated inhibitors for 24 h. Afterwards, A total of 10^6^ cells were collected and fixed overnight in 70% ethanol. The fixed cells were washed with PBS 3 times, stained with PI (BD-Pharmingen, United States) for 15 min, and analyzed using a flow cytometer (Beckman Coulter, Shanghai). The analysis of cell cycle was performed using ModFit LT 5.0 software.

### Western blot analysis

Cells were lysed in RIPA buffer (Beyotime, China) with protease and phosphatase inhibitor cocktails (Beyotime, China). Proteins separated by SDS-PAGE were transferred onto PVDF membranes and blocked in 3% bovine serum. Afterwards, membranes were incubated overnight in primary antibody solution at 4°C, followed by incubation with an HRP-conjugated secondary antibody for 1 h at room temperature. Band signals were detected using an Amersham Imager 600 (General Electric Company, Boston, MA, United States), and quantification was performed using ImageJ software.

### RNA-seq

Total RNA was extracted using TRIzol reagent (Invitrogen) according to the manufacturer’s instructions. RNA-seq data from cell lines were generated using 2*100 bp paired-end sequencing using the Illumina HiSeq 2000 platform. Paired-end reads were mapped to the University of California at Santa Cruz (UCSC) hg19 reference genome using TopHat2. The normalized expression level of each gene is expressed as the fragments per kilobase of transcript per million mapped reads (FPKM) value. Differential expression analysis of two cell lines (3 biological replicates) was performed using the DESeq2 R package (1.16.1). The resulting P-values were adjusted using Benjamini and Hochberg’s approach for controlling the false discovery rate. Genes with an adjusted P-value < 0.05 found by DESeq2 were assigned as differentially expressed.

### RNA extraction and quantitative real-time PCR

Total RNA was extracted according to the procedure of the RNeasy kit (Qiagen, Canada). cDNA from each sample was reverse transcribed using the PrimeScript RT Master Mix Kit (Takara, Japan). Quantitative PCR was performed on cDNA using the SYBR Green System (Applied Biosystems). GAPDH was used as an endogenous control. The relative quantification of RT-qPCR data was calculated using the 2−ΔΔCT method as described previously.

### Statistics

Data are presented as the mean ± standard error of the mean (SEM) or standard deviation (SD). Statistical analysis was conducted using Prism 8.0 (GraphPad Software, San Diego, CA). Statistical analyses were performed with the chi-square test, Student’s t-test, and one-way analysis of variance (ANOVA), as appropriate. P-values < 0.05 were considered to indicate statistical significance, and asterisks (*) are used to indicate significant differences between two specified groups. ** indicates a P-value < 0.01, while *** indicates a P-value < 0.001. P-values > 0.05 qualified as not statistically significant.

## Results

### Establishment of MEKi-resistant MPNST cell lines

**Figure 1:**
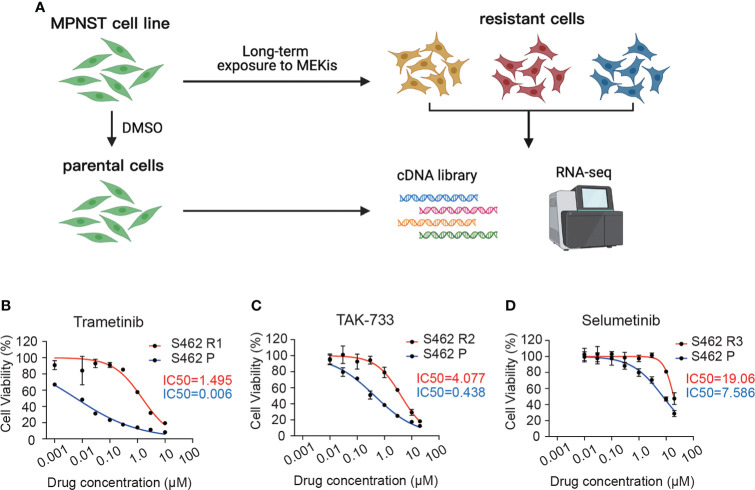
Establishment and verification of MEKi-resistant MPNST cell lines. **(A)** Schematic representation of developing MEKi-resistant MPNST cell models. **(B–D)** Cell viability assays of three MEKi-resistant cell lines and parental cells treated with trametinib **(B)**, TAK-733 **(C)**, and selumetinib **(D)**. All experiments were performed in triplicate, and each point represents Mean ± SEM. S462 P: S462 parental cells, S462 R1: S462 cells resistant to trametinib, S462 R2: S462 cells resistant to TAK-733, S462 R3: S462 cells resistant to selumetinib.  .

To investigate mechanisms relevant to MEKi resistance and to explore effective targets for combination therapy, we generated 3 S462 cell lines resistant to trametinib (S462 R1), TAK-733 (S462 R2), and selumetinib (S462 R3) *via* continuous exposure of the MEKi-sensitive cell line (S462) to either vehicle or different MEKis (**Figure 1A**). The drug resistance of MEKi-resistant cell lines was verified by CCK-8 assays after withdrawal of MEKis for over 2 months (**Figures 1B–D**, **Figure S1**). The results showed that the cell viability, as well as the IC50 value, significantly increased following the acquisition of drug resistance in S462 cells.

### The cell-cycle arrest and apoptosis induced by MEKis were rescued in MEKi-resistant MPNST cell lines

**Figure 2:**
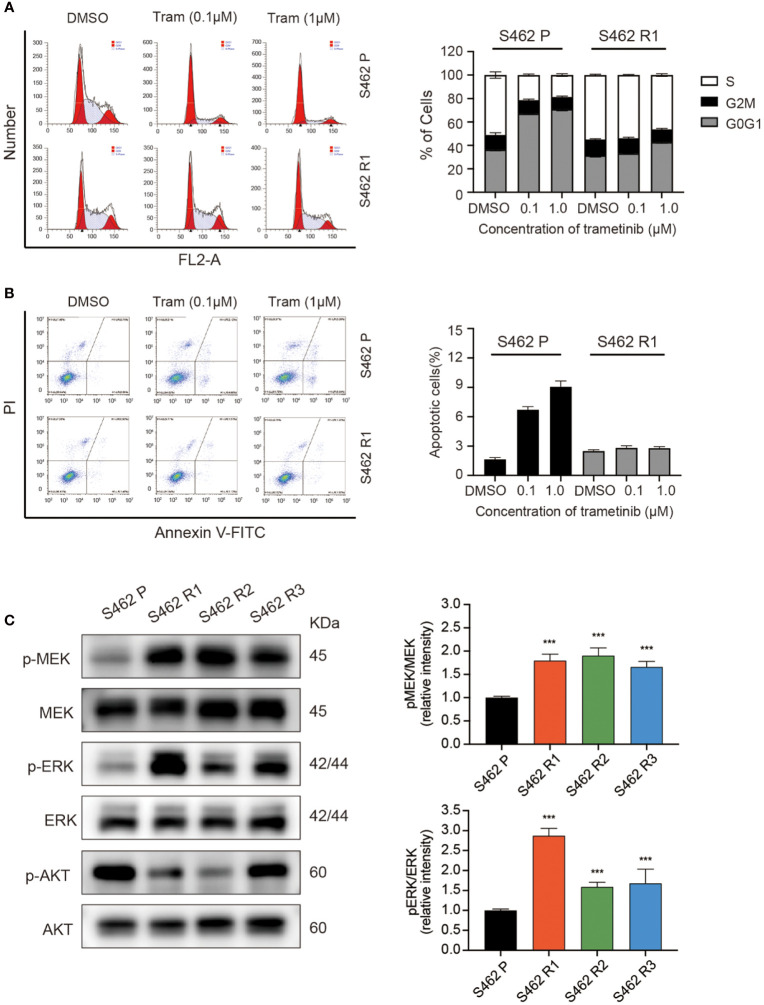
MEKi-induced cell-cycle arrest and apoptosis were rescued in MEKi-resistant MPNST cell lines. **(A)** cell cycle assays and **(B)** Annexin V–FITC and propidium iodide (PI) assays of S462 R1 and S462 P cells upon trametinib or vehicle treatment for 24 h. All experiments were performed in triplicate, and each column represents Mean ± SEM. **(C)** Expression of p-MEK, MEK, p-ERK, and ERK in S462 P, S462 R1, S462 R2, and S462 R3 cells evaluated by western blot. All experiments were performed in triplicate, and each column represents Mean ± SEM. ***p < 0.001. S462 P: S462 parental cells, S462 R1: S462 cells resistant to trametinib, S462 R2: S462 cells resistant to TAK-733, S462 R3: S462 cells resistant to selumetinib.

It was demonstrated in previous studies that MEKis exerted anti-proliferative effects *via* induction of cell apoptosis and cell cycle arrest. Therefore, following the validation of the resistant cell population, we set out to characterize the differences in cell cycle and cell apoptosis in MEKi-resistant and parental cells exposed to MEKis. As demonstrated in the flow cytometry analysis, the G0/G1 phase blockade induced by trametinib was rescued in S462 R1 cells (**Figure 2A**). Similarly, the ratios of apoptotic cells significantly decreased in S462 R1 cells compared to S462 parental cells (**Figure 2B**). In addition, the upregulation of cleaved PARP expression after trametinib treatment was less obvious in S462 R1 cells compared to S462 parental cells (**Figure S2**), indicating that the survival and proliferation capacity of MPNST cells significantly improved after the acquisition of resistance to MEKis. Since the previous studies showed that the reactivation of MAPK pathway or the compensatory activation of parallel pathway was the mechanisms mainly contributing to MEKi resistance (20, 29), we next evaluated the MEK/ERK and AKT activities in MEKi-resistant cell lines (S462 R1, S462 R2, S462 R3). The expression of p-MEK and p-ERK was upregulated in 3 MEKi-resistant cell lines compared with S462 P, while the expression of P-AKT/AKT did not increase (**Figure 2C**).

### Dysregulation of focal adhesion pathway is associated with MEKi resistance in MPNST

**Figure 3:**
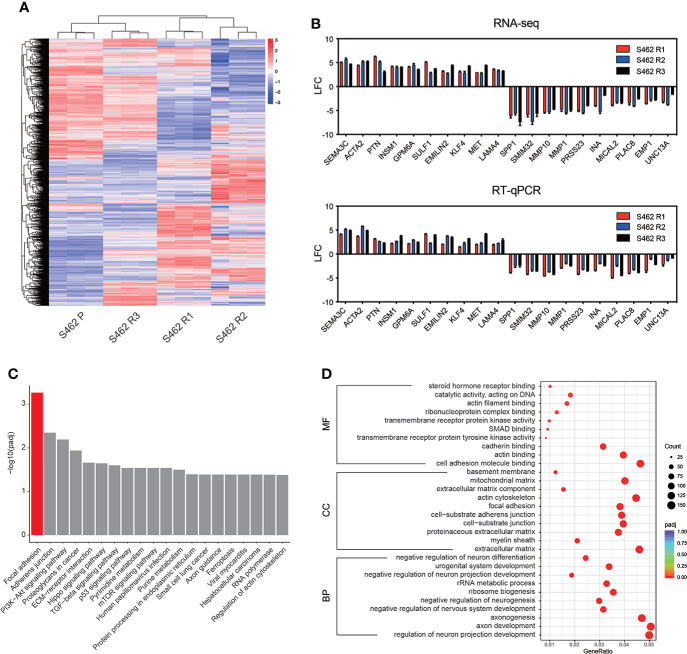
Focal adhesion signaling pathway is dysregulated in MEKi-resistant MPNST cell lines. **(A)** RNA-seq heat map showing the differences of gene expression in 3 MEKi-resistant cell lines. The S462 parental cell line served as a control (blue: decreased expression, red: increased expression). **(B)** The expression of the top 10 upregulated and downregulated genes was confirmed by PCR analysis. All experiments were performed in triplicate, and each column represents Mean ± SEM. **(C, D)** KEGG **(C)** and GO enrichment **(D)** analysis of the common DEGs in MEKi-resistant MPNST cell lines S462R1, S462 R2, and S462 R3. S462 P: S462 parental cells, S462 R1: S462 cells resistant to trametinib, S462 R2: S462 cells resistant to TAK-733, S462 R3: S462 cells resistant to selumetinib.

To investigate mechanisms contributing to MEKi resistance in MPNSTs, high-throughput transcriptomic sequencing of 3 MEKi-resistant cell lines (S462 R1, S462 R2, and S462 R3) was conducted, and the S462 P cell line served as a control. The overall differences in gene expression at transcriptional level were visualized with the heatmap (**Figure 3A**). The common differentially expressed genes (DEGs) in 3 MEKi-resistant cell lines were demonstrated and verified by RT-qPCR in **Figure 3B**. In addition, gene set enrichment analysis in the Kyoto Encyclopedia of Genes and Genomes (KEGG) and Gene Ontology (GO) identified signaling pathways that significantly dysregulated in MEKi-resistant MPNST cells. Both KEGG and GO analysis identified significant activation of focal adhesion signaling pathway in three MEKi-resistant MPNST cell models (**Figures 3C**, **D**, **Figure S3**).

### LAMA4 induced MEKi resistance by upregulating integrin b1/FAK/SRC signaling in MPNST

**Figure 4:**
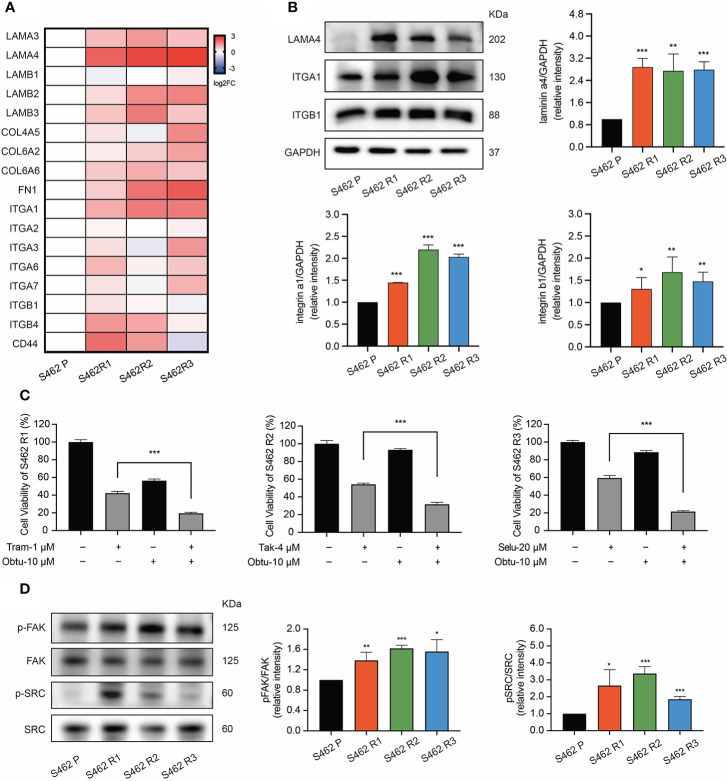
The upregulation of laminin/integrin/FAK/SRC axis mediated MEKi resistance in MPNSTs. **(A)** RT-qPCR analysis of the DEGs related to focal adhesion pathway in S462 P, S462 R1, S462 R2, and S462 R3 cells. All experiments were performed in triplicate. **(B)** western blot analysis of f LAMA4, ITGA1, and ITGB1 in S462 P, S462 R1, S462 R2, and S462 R3 cells. All experiments were performed in triplicate, and each column represents Mean ± SEM. *p < 0.05, **p < 0.01, ***p < 0.001. **(C)** Cell viability of S462 R1 exposed to DMSO, trametinib (Tram), obtustatin (Obtu) or combination therapy, cell viability of S462 R2 exposed to DMSO, TAK-733 (Tak), obtustatin or combination therapy, and cell viability of S462 R3 exposed to DMSO, selumetinib (Selu), obtustatin or combination therapy. All experiments were performed in triplicate, and each column represents Mean ± SEM. *p < 0.05, **p < 0.01, ***p < 0.001. **(D)** Expression of p-FAK, FAK, p-SRC, and SRC in S462 P, S462 R1, S462 R2, and S462 R3 cells was evaluated by western blot. All experiments were performed in triplicate, and each column represents Mean ± SEM. *p < 0.05, **p < 0.01, ***p < 0.001. S462 P: S462 parental cells, S462 R1: S462 cells resistant to trametinib, S462 R2: S462 cells resistant to TAK-733, S462 R3: S462 cells resistant to selumetinib.

RT-qPCR analysis was performed to explore the expression of the DEGs related to focal adhesion signaling in 3 MEKi-resistant cell lines and parental cells. The results showed that the top gene significantly upregulated was LAMA4, whose receptors including ITGA1 and ITGB1 were also overexpressed (**Figure 4A**). Western blot analysis further confirmed that the protein levels of these genes were upregulated in MEKi-resistant cell lines (**Figure 4B**). Furthermore, inhibiting integrinα1β1 using the highly selective inhibitor obtustatin increased the sensitivity to MEKis according to cell viability assays (**Figure 4C**), indicating that the upregulation of LAMA4/ITGB1 axis contributed to MEKi resistance. Based on these results, we further evaluated the expression of FAK/SRC, the key regulatory molecules of integrin signaling, and its downstream pathways (**Figure 4D**). According to western blot analysis, the phosphorylation of FAK and SRC was significantly increased in 3 MEKi-resistant cell lines (S462 R1, S462 R2, S462 R3) compared with S462 parental cells (S462 P). Altogether, these data suggested that the upregulation of the laminin/integrin/FAK/SRC axis could reactivate MAPK signaling, leading to MEKi resistance.

### Concurrent inhibition of MAPK signaling and FAK/SRC cascade partially overcame MEKi resistance in MPNST cell lines

**Figure 5:**
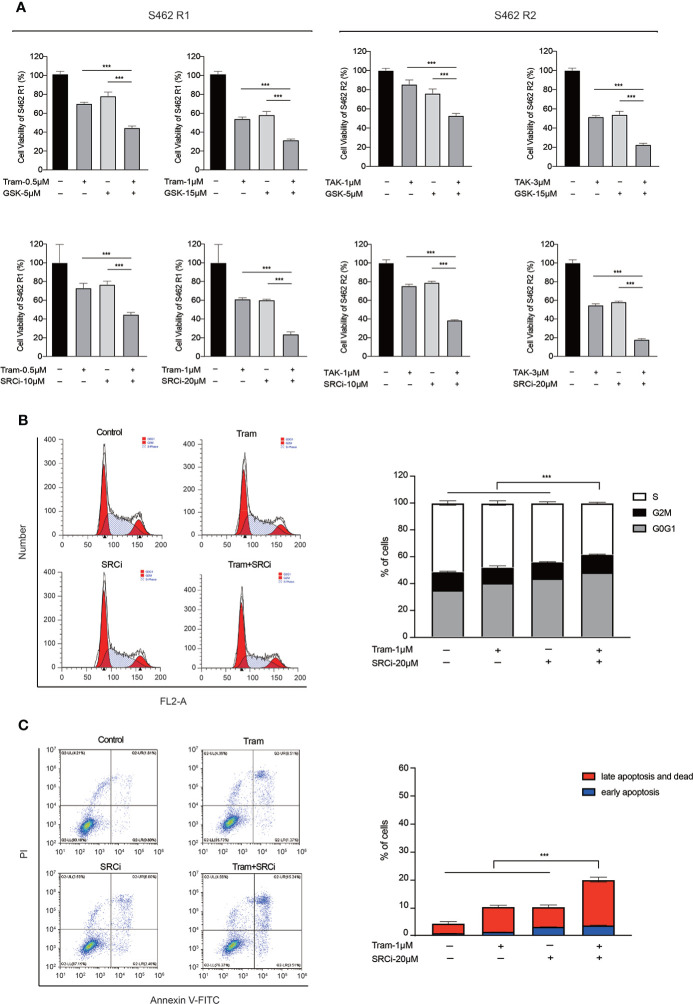
Concurrent inhibition of MAPK signaling and FAK/SRC cascade partially overcame MEKi resistance in MPNST cell lines. **(A)** Cell viability of S462 R1 exposed to DMSO, trametinib (Tram), GSK2256098 (GSK), SRC inhibitor 1 (SRCi), or combination therapy and cell viability of S462 R2 exposed to DMSO, TAK-733 (TAK), GSK2256098 (GSK), SRC inhibitor 1 (SRCi) or combination therapy. All experiments were performed in triplicate, and each column represents Mean ± SEM. ***p < 0.001. **(B)** Cell cycle and **(C)** apoptosis ratios of S462 R1 treated with DMSO, trametinib (Tram), SRC inhibitor 1 (SRCi), or combination therapy. All experiments were performed in triplicate, and each column represents Mean ± SEM. ***p < 0.001. S462 P: S462 parental cells, S462 R1: S462 cells resistant to trametinib, S462 R2: S462 cells resistant to TAK-733.

Since our findings were consistent with previous studies showing that FAK/SRC conferred resistance to targeted therapies in a variety of solid tumors (30), we hypothesized that targeting the FAK/SRC signaling is a potential therapeutic strategy to overcome resistance to MEKis. We tested FAK inhibitor GSK2256098 and SRC inhibitor 1, as single agents and in combination with MEKis in S462 R1, and S462 R2 (**Figure 5A**, **Figures S4**, **5**). According to CCK-8 assays, we found that combinational inhibition of FAK/SRC and MEK had greater effects on cell viability than single agents. In addition, the S-phase arrest and apoptosis induced by SRC inhibitor 1 and trametinib combination therapy were much more significant than single agents in S462 R1 cells (**Figures 5B**, **C**). The results above indicated that concurrent inhibition of MAPK signaling and FAK/SRC cascade could restore the sensitivity of MPNST cells to MKEis.

### MEK and SRC inhibitors showed combination effect in MPNST cell models

**Figure 6:**
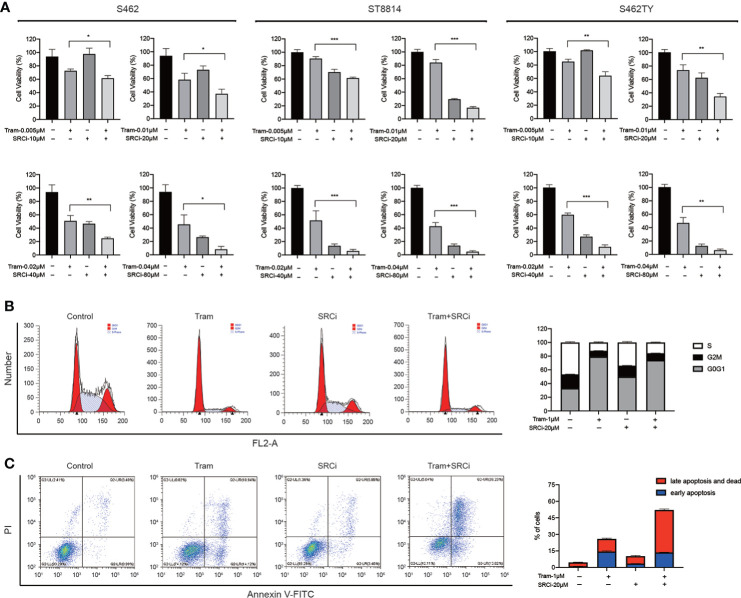
The efficacy of combined MEKi and SRCi therapy in MPNSTs. **(A)** Cell viability of S462, ST8814, and S462TY exposed to DMSO, trametinib (Tram), SRC inhibitor 1 (SRCi), or combination therapy. All experiments were performed in triplicate, and each column represents Mean ± SEM. *p < 0.05, **p < 0.01, ***p < 0.001. **(B)** Cell cycle and **(C)** apoptosis ratios of S462 treated with DMSO, trametinib (Tram), SRC inhibitor 1 (SRCi), or combination therapy. All experiments were performed in triplicate, and each column represents Mean ± SEM. *p < 0.05, **p < 0.01, ***p < 0.001.

Having shown that FAK/SRC inhibitors sensitized resistant cell lines to MEKis, we next investigated whether combined MEK/SRC inhibition could reduce cell growth in MEKi-sensitive MPNST cells. The CCK-8 assays showed that trametinib and SRC inhibitor 1 had only modest activity as single agents in MPNST cell lines ST8814, S462, and S462TY, but the combination therapy had a more profound effect on cell viability compared to either drug alone (**Figure 6A**, **Figure S6**). To quantify drug synergy between trametinib and SRC inhibitor 1, we calculated combination index (CI) values using Compusyn software. The CI values for S462, ST8814, and S462TY cells are 0.52, 0.44, and 0.62, respectively, indicating the synergistic effects between trametinib and SRC inhibitor 1 in MPNST cells.

To explore the mechanism underlying the synthetic lethality, we conducted cell cycle and apoptosis assays. The results revealed that the combination therapy induced cell apoptosis much more robust than single agents in S462 cells. According to western blot assays, SRC inhibitor 1 could not upregulate cleaved PARP expression in S462 cells but can significantly increase the activation of cleaved PARP induced by MEKi trametinib. However, the cell cycle arrest was not increased in S462 cells treated with combination therapy (**Figures 6B**, **C**, **Figure S7**). These results indicated that the combination effect of MEK and SRC inhibitors was caused by the induction of apoptosis in MPNSTs.

## Discussion

**Figure 7:**
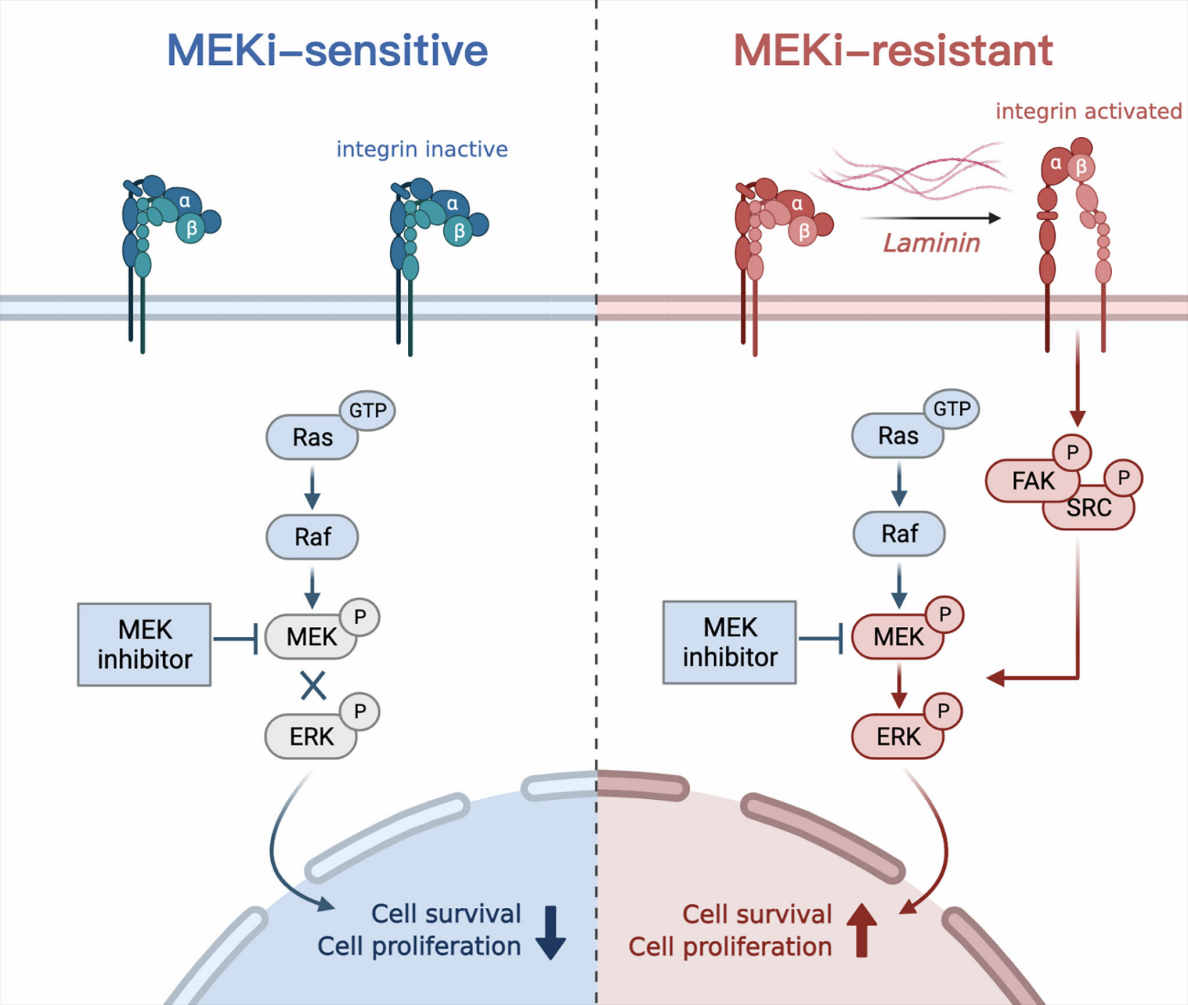
Schematic of the study. LAMA4 induced MEKi resistance in MPNSTs by upregulating integrin b1/FAK/SRC pathway and subsequently reactivating MAPK signaling.

In this study, we investigated mechanisms of MEKi resistance in MPNSTs using MEKi-resistant cell models and high-throughput transcriptomic sequencing. Our study identified the upregulation of LAMA4/ITGB1/FAK/SRC axis and subsequent reactivation of MAPK pathway as causative factors that mediated resistance to MEKis. In addition, we demonstrated the effectiveness of combined FAK/SRC and MEK inhibition in enhancing the response of both resistant and sensitive MPNST cells to MEKis (**Figure 7**).

Hyperactivation of RAS is a key oncogenic event contributing to many cancers including MPNST. Although targeting its downstream MAPK signaling pathway was considered a promising strategy, the efficacy of MEKi monotherapy was insufficient for the treatment of MPNSTs (31, 32). To improve the clinical effect of MEKis and to develop effective targeted therapeutic strategies for MPNSTs, mechanisms of MEKi resistance need to be clarified. As shown in previous investigations, tumors evade targeted cancer therapies *via* an extensive repertoire of resistance mechanisms. The mechanisms of MEKi resistance that have been identified include, but are not limited to, reactivation of the MAPK pathway, activation of parallel signaling pathways, dysregulation of transcription factors, and transformation in cellular phenotype (29, 33–35). Specifically, Wang et al. discovered that HGF overexpression conferred MEKi resistance by re-activating both PI3K/AKT and MAPK signaling in MPNSTs (25). Our team identified the activation of cyclin-dependent kinase signaling as the vulnerability to overcome MEKi resistance in pNF (36). In this study, we verified the reactivation of MAPK signaling in various MEKi-resistant cell models, accompanied by enhanced abilities to survive and proliferate against MEKis. Previous studies also verified the importance of MAPK reactivation in MEKi resistance and revealed various mechanisms that led to ERK activation, including alterations to molecules upstream of ERK such as RAF, mutation of MEK, and reactivation of multiple RTKs upstream of the MAPK pathway (37–41). These findings suggested that targeting the mechanisms of MAPK reactivation could be an effective strategy to overcome MEKi resistance in MPNSTs.

In this study, we identified that the upregulation of LAMA4/ITGB1/FAK/SRC signaling conferred reactivation of MAPK signaling and resistance to MEKis using high-throughput transcriptomic sequencing. Laminin α4 and its receptor β1 integrin play important roles in mediating cell adhesion and mechanochemical signaling (42, 43). Recent studies revealed that aberrant expression of laminin/integrin signaling was involved in mediating tumor resistance to chemotherapy and targeted therapies (44–46), termed cell adhesion-mediated drug resistance (CAMDR). For example, it was demonstrated in ErbB2-positive breast cancer that laminin and integrins a6b4, a3b1 could lead to acquired resistance to lapatinib and trastuzumab through mitogenic and pro-survival signaling (47). In addition, a previous study also verified that laminin/integrin signaling could promote the survival and proliferation of MPNST tumorigenic cells (48). These findings highlight the important role of laminin/integrin signaling in MEKi resistance.

As the key downstream mediator of the integrin signaling pathway, focal adhesion kinase (FAK), also has an important role in mediating drug resistance (49–52). Phosphorylated FAK forms a complex with SRC, which activates downstream signaling pathways such as PI3K/Akt, MAPK, and Rho, promoting the proliferation, survival, and protein synthesis of tumor cells (53, 54). Our study demonstrated that the phosphorylation of FAK/SRC also significantly increased in MEKi-resistant MPNST cells. In addition, concurrent inhibition of FAK/SRC and MAPK signaling reversed MEKi resistance by inducing apoptosis and cell cycle arrest. These observations were consistent with previous studies which also verified that targeting FAK/SRC sensitized drug-resistant tumor cells to targeted therapies (55). In breast cancer, inhibition of FAK expression with small-molecule inhibitors significantly increased the sensitivity of breast cancer cells to the ErbB2 antibody trastuzumab (56). Meanwhile, in a mouse model of orthotopic glioblastoma, blocking FAK autophosphorylation promoted temozolomide-induced cell death (57). It was also demonstrated that SRC small molecule inhibitor overcame the MAPKi and PI3K/mTORi dual-drug resistance in melanoma (58).

Targeting the key pathways of drug resistance is an effective way to improve the sensitivity of cancers, which is of great significance for improving the survival rate of patients with advanced tumors (18, 59). For example, in BRAF-mutated malignant melanoma, the combination of BRAF inhibitor encorafenib and MEK inhibitor binimetinib was more effective than single agents, showing significant effect in delaying the onset of drug resistance and improving the overall survival rate of patients (60, 61). Therefore, we speculate that the combination therapy targeting FAK/SRC and MEK could be an effective therapeutic strategy for MPNSTs. We investigated the efficacy of combination therapy with SRC and MEK inhibitors in several MPNST cell models and found out that combined MEK/SRC inhibitor treatment exerted synthetic lethality by inducing cell apoptosis in MPNST. At present, there are a few clinical trials evaluating the efficacy of combination therapy with FAK/SRC and MEK inhibitors. A phase I clinical trial also revealed that the combination of FAK inhibitor defactinib and RAF/MEK inhibitor VS-6766 achieved promising results in low-grade serous ovarian cancer (62). Therefore, co-targeting FAK/SRC and MEK might be an effective strategy for the treatment of MPNSTs, which is worth exploring in the clinic.

In conclusion, we discovered that the upregulation of integrin signaling mediated resistance to MEKis. Combinational inhibition of FAK/SRC and MAPK signaling was an effective strategy to overcome MEKi resistance and improve the efficacy of MEKis in MPNSTs. Further clinical investigations that combine MEK and FAK/SRC inhibitors in MPNSTs should be considered in the future.

## Data availability statement

The datasets presented in this study can be found in online repositories. The names of the repository/repositories and accession number(s) can be found below: https://www.ncbi.nlm.nih.gov/geo/, GSE174100.

## Author contributions

YG, CW, MC and HL: Conceptualization, Investigation. ZG, ML, YL and RA: Formal analysis, Visualization. WW and YG: Writing; QL and ZW: Supervision. All authors contributed to the article and approved the submitted version.

## Funding

This work was supported by grants from National Natural Science Foundation of China (82102344; 82172228); Shanghai Rising Star Program supported by Science and Technology Commission of Shanghai Municipality (20QA1405600); Science and Technology Commission of Shanghai Municipality (19JC1413); Natural Science Foundation of Shanghai (22ZR1422300); “Chenguang Program” supported by Shanghai Education Development Foundation (SHEDF) (19CG18); Shanghai Municipal Key Clinical Specialty (shslczdzk00901); Innovative research team of high-level local universities in Shanghai (SSMU-ZDCX20180700); the Project of Biobank (YBKA201901) from Shanghai Ninth People’s Hospital, Shanghai Jiao Tong University School of Medicine; Shanghai Anticancer Association (SHCY-JC-2021114).

## Acknowledgments

The authors would like to thank Prof. Vincent Keng and Prof. Jilong Yang for providing cell lines. Figures were created using Adobe illustrator 2019 and Biorender.

## Conflict of interest

The authors declare that the research was conducted in the absence of any commercial or financial relationships that could be construed as a potential conflict of interest.

## Publisher’s note

All claims expressed in this article are solely those of the authors and do not necessarily represent those of their affiliated organizations, or those of the publisher, the editors and the reviewers. Any product that may be evaluated in this article, or claim that may be made by its manufacturer, is not guaranteed or endorsed by the publisher.
