# Supplementary material for: Concurrent inhibition of FAK/SRC and MEK overcomes MEK inhibitor resistance in Neurofibromatosis Type I related malignant peripheral nerve sheath tumors
Source: Front Oncol. 2022 Jul 29;12:910505. doi: 10.3389/fonc.2022.910505 (PMC9372505; doi:10.3389/fonc.2022.910505)
Supplement: Supplementary file 1 [file DataSheet_1.docx]

Supplementary Material

Table S1. Reagents and antibodies

| Product Name | Company | Cat.No. |
| --- | --- | --- |
| trametinib | MedChemExpress | HY-10999 |
| TAK-733 | MedChemExpress | HY-13449 |
| selumetinib | MedChemExpress | HY-50706 |
| GSK2256098 | MedChemExpress | HY-100498 |
| SRC inhibitor 1 | MedChemExpress | HY-101053 |
| ERK1/2 antibody | CST | 4695 |
| p-ERK antibody | CST | 4370 |
| MEK1/2 antibody | CST | 4694 |
| p-MEK antibody | CST | 2338 |
| AKT antibody | CST | 4691S |
| p-AKT antibody | CST | 4060 |
| FAK antibody | CST | 3285 |
| p-FAK antibody | CST | 8556 |
| SRC antibody | CST | 2109T |
| p-SRC antibody | CST | 6943T |
| GAPDH antibody | EASYBIO | BE0034 |
| ITGA1 antibody | Santa Cruz | Sc-271034 |
| ITGB1 antibody | abcam | Ab134179 |
| LAMA4 antibody | Abclonal | A15286 |
| Obtustatin | AdooQ | A17185 |


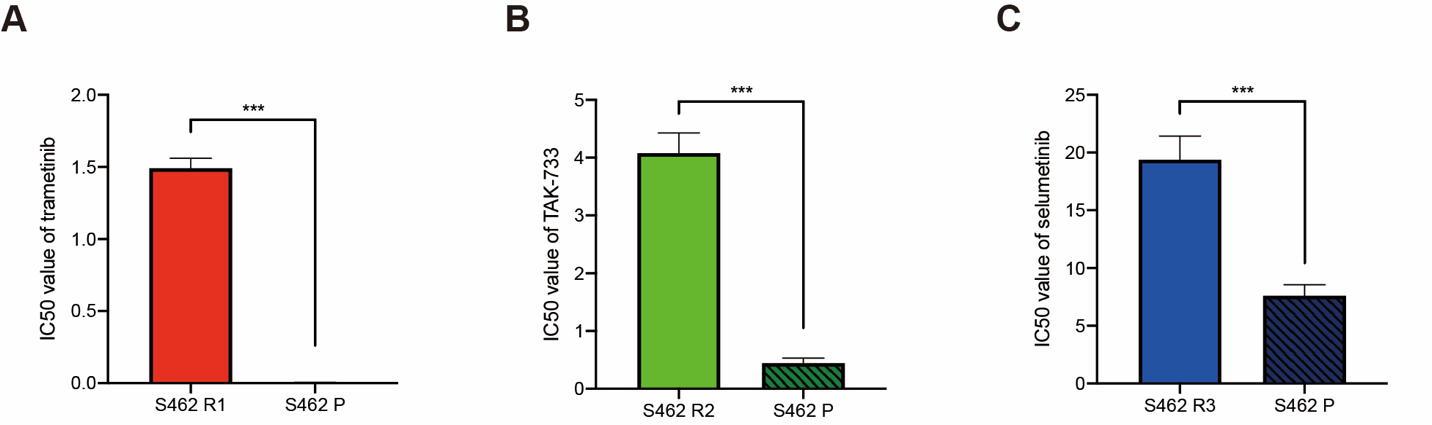


Figure S1. IC50 value of trametinib in S462 R1 (A), TAK-733 in S462 R2 (B) and selumetinib in S462 R3 (C). All experiments were performed in triplicate, and each column represents Mean ± SEM. ***p < 0.001. S462 P: S462 parental cells, S462 R1: S462 cells resistant to trametinib, S462 R2: S462 cells resistant to TAK-733, S462 R3: S462 cells resistant to selumetinib.


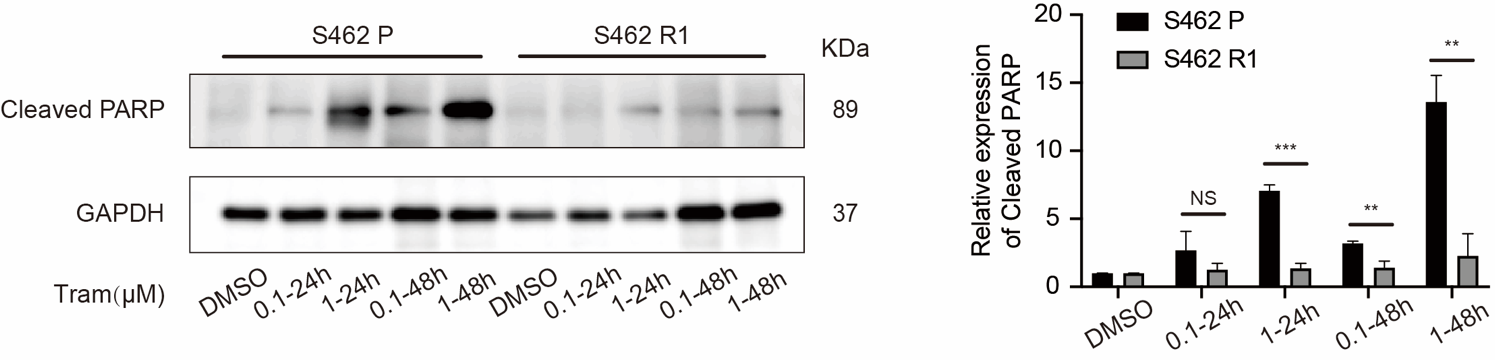


Figure S2. Cleaved PARP expression in S462 P and S462 R1 exposed to different concentrations of trametinib for 24h or 48h. All experiments were performed in triplicate, and each column represents Mean ± SEM. **p < 0.01, ***p < 0.001. S462 P: S462 parental cells; S462 R1: S462 cells resistant to trametinib.


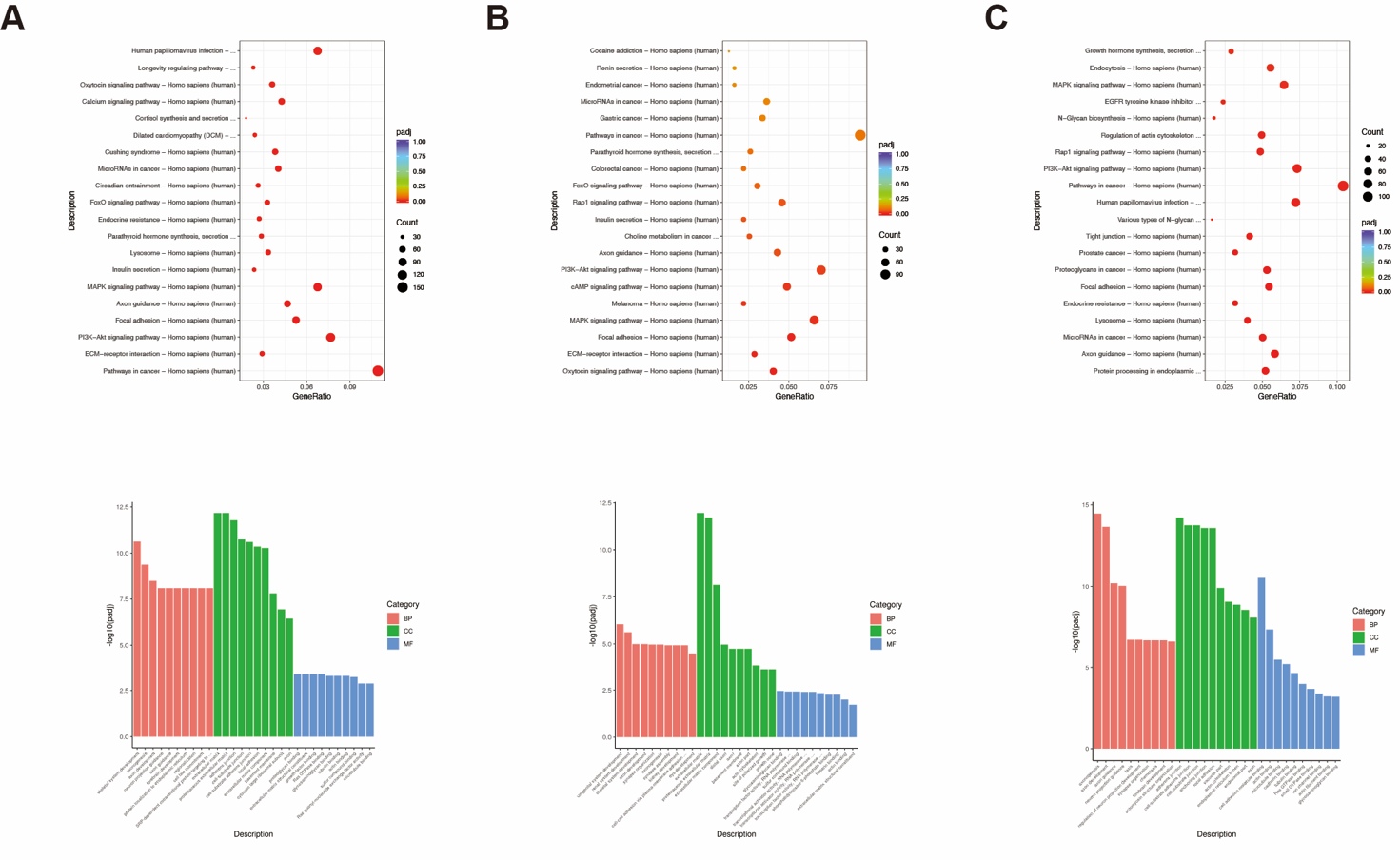


Figure S3. KEGG and GO enrichment analysis of upregulated DEGs in MEKi-resistant MPNST cell lines S462R1 (A), S462 R2(B), S462 R3(C). S462 R1: S462 cells resistant to trametinib, S462 R2: S462 cells resistant to TAK-733, S462 R3: S462 cells resistant to selumetinib.


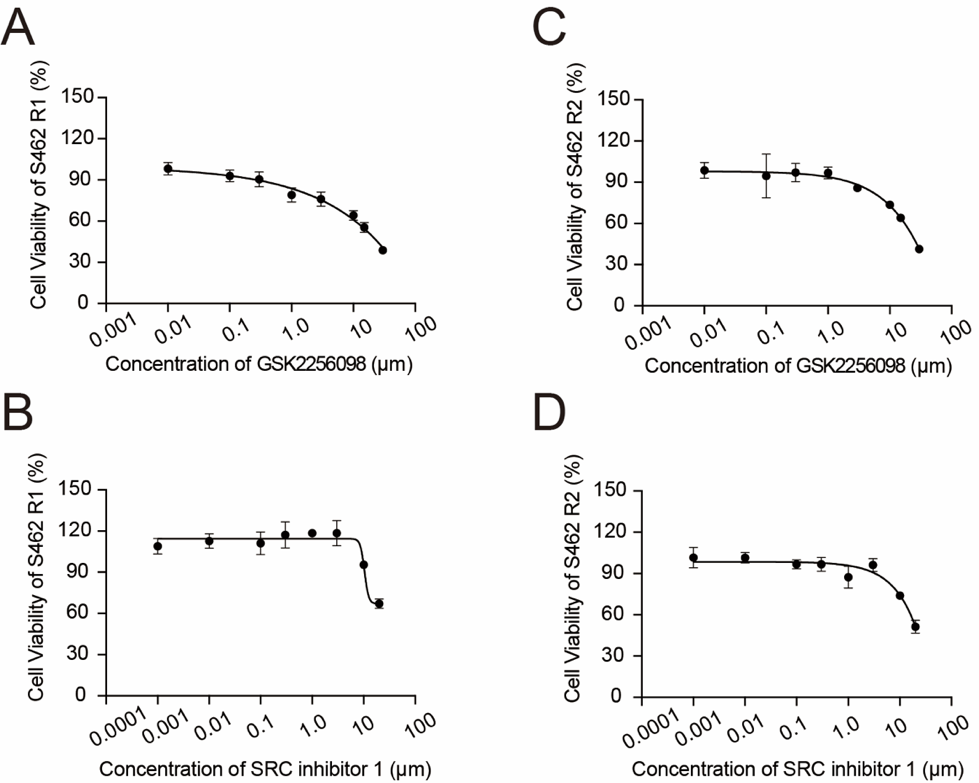


Figure S4. (A-B) Cell viability of S462 R1 exposed to different concentrations of GSK2256098(A) and SRC inhibitor 1(B). (C-D) Cell viability of S462 R2 exposed to different concentrations of GSK2256098(C) and SRC inhibitor 1(D). All experiments were performed in triplicate, and each point represents Mean ± SEM. S462 R1: S462 cells resistant to trametinib, S462 R2: S462 cells resistant to TAK-733.


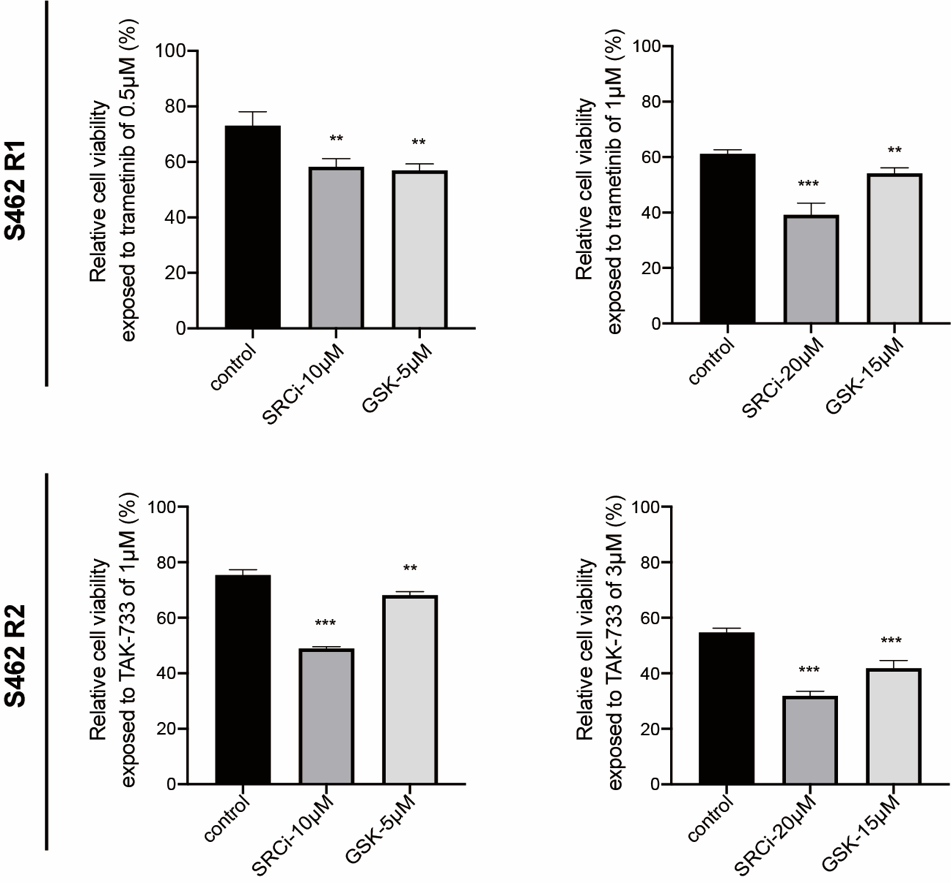


Figure S5. Cell viability of S462 R1 exposed to trametinib (Tram) monotherapy or in combination with GSK2256098 (GSK) and SRC inhibitor 1 (SRCi) and cell viability of S462 R2 exposed to TAK-733 (TAK) monotherapy or in combination with GSK2256098 (GSK) and SRC inhibitor 1 (SRCi). All experiments were performed in triplicate, and each column represents Mean ± SEM. ***p* < 0.01, ****p* < 0.001.


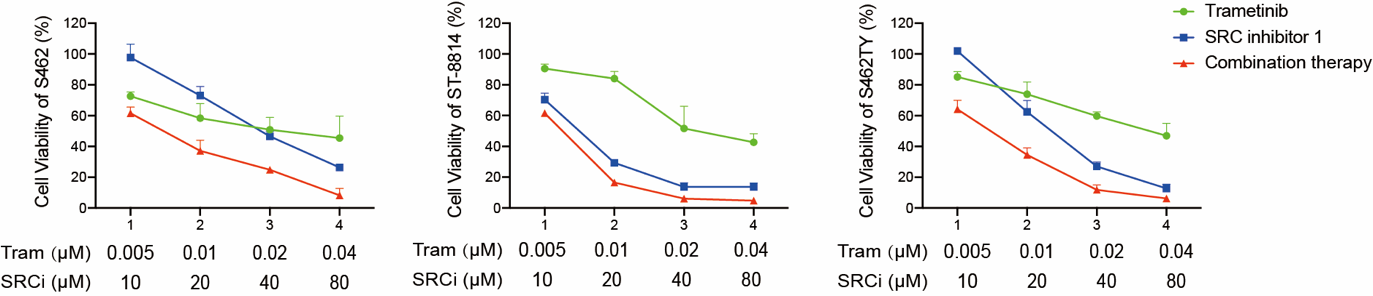


Figure S6. Cell viability of S462, ST8814, and S462TY exposed to DMSO, trametinib (Tram), SRC inhibitor 1 (SRCi), or combination therapy. All experiments were performed in triplicate, and each point represents Mean ± SEM.


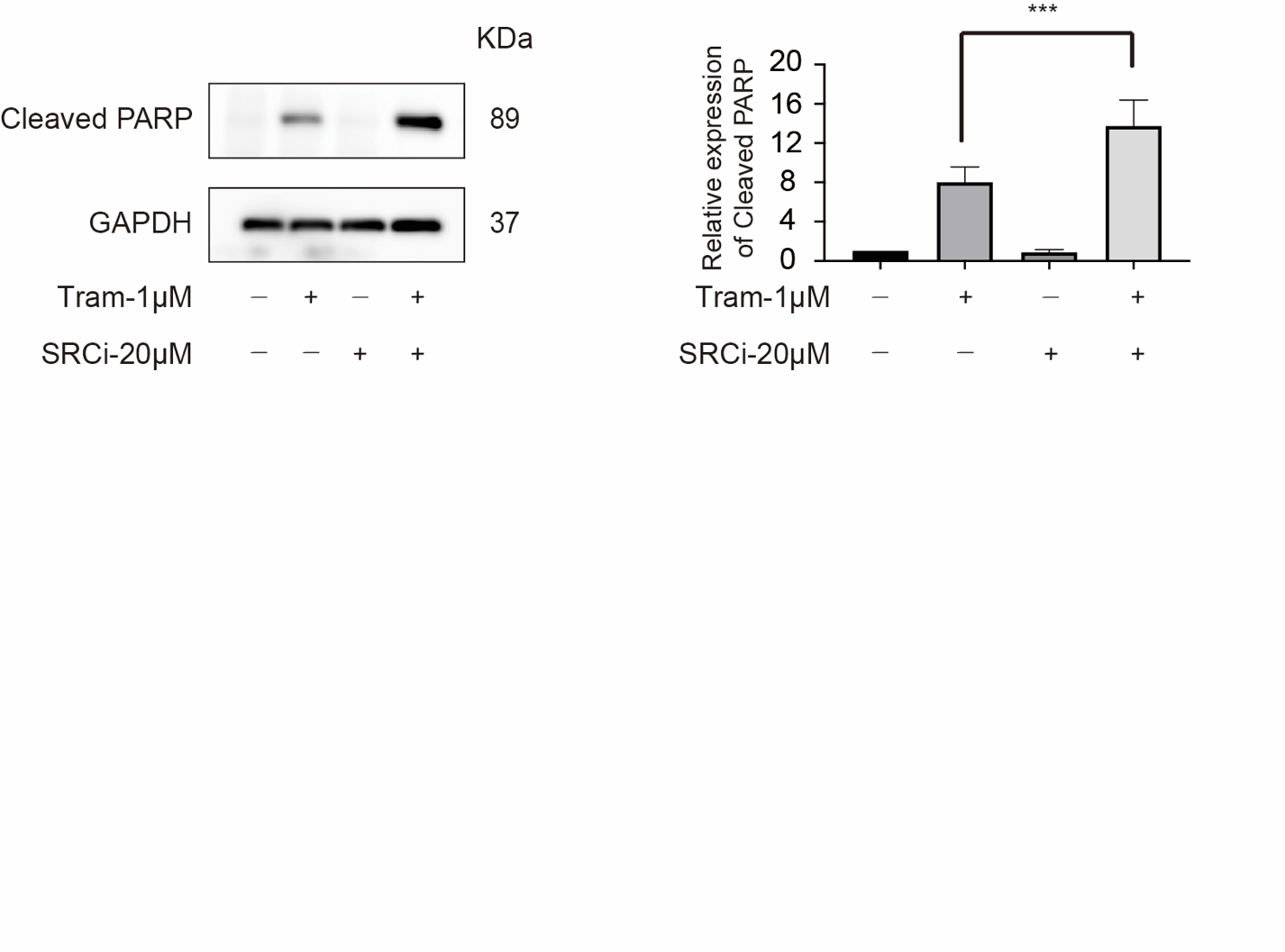


Figure S7. Cleaved PARP expression in S462 cells exposed to trametinib (Tram) or/and SRC inhibitor 1 (SRCi) for 24h. All experiments were performed in triplicate, and each column represents Mean ± SEM. ***p < 0.001. S462 P: S462 parental cells; S462 R1: S462 cells resistant to trametinib.
